# Sleep and Inflammation during COVID-19 Virtual Learning in Adolescents with Overweight or Obesity

**DOI:** 10.3390/children10121833

**Published:** 2023-11-21

**Authors:** Lindsay M. Stager, Casie H. Morgan, Caroline S. Watson, Aaron D. Fobian

**Affiliations:** 1 Department of Psychology, University of Alabama at Birmingham, 1300 University Blvd, Birmingham, AL 35233, USA; lmstager@uab.edu (L.M.S.); chmorgan@uab.edu (C.H.M.); carolineswatson@uabmc.edu (C.S.W.); 2 Department of Psychiatry and Behavioral Neurobiology, University of Alabama at Birmingham, 1720 7th Avenue South, Birmingham, AL 35233, USA

**Keywords:** COVID-19, adolescent, sleep, inflammation, obesity

## Abstract

(1) Background: Adolescents present as a high-risk group for a range of adverse physical health outcomes during the pandemic, including sleep and C-reactive protein (CRP) levels. As adolescents with overweight or obesity (OWOB) present as an even higher risk group, the present study assessed relationships between sleep and CRP levels before and during COVID-19 in adolescents with OWOB. (2) Methods: Fourteen adolescents with OWOB participated in a pre-COVID1, pre-COVID2, and during-COVID-19 lab visit, measuring sleep and CRP levels. The sample size was limited by the number of participants who provided data before COVID-19 and who were enrolled in virtual school during the recruitment phase. However, our power analyses indicated needing a minimum of 10 participants to achieve adequate power. Pre-COVID1, pre-COVID2, and during-COVID-19 normative expected CRP levels were calculated based on age, sex, race, and body mass index percentile-matched data. Analyses compared pre-COVID1 and pre-COVID2 sleep with during-COVID-19 sleep, during-COVID-19 sleep and during-COVID-19 CRP levels, during-COVID-19 CRP levels with normative expected during-COVID-19 CRP levels, change in CRP levels from pre-COVID1 and pre-COVID2 to during-COVID-19 with normative expected CRP levels during those time periods, and change in CRP levels before COVID-19 with change in CRP levels during COVID-19. (3) Results. During COVID-19, participants experienced decreased sleep efficiency (*p* = 0.001), later wake time (*p* < 0.001), longer time in bed (*p* = 0.021), and onset latency (*p* = 0.004), compared to pre-COVID1, and decreased sleep efficiency (*p* = 0.002), longer onset latency (*p* = 0.006), and later wake time (*p* < 0.001) and bedtime (*p* = 0.016) compared with pre-COVID2. During-COVID-19 CRP levels were positively correlated with during-COVID-19 wake times (*p* = 0.01) and times in bed (*p* = 0.008). During-COVID-19 CRP levels were greater than normative expected CRP levels (*p* < 0.001). CRP levels increased more from pre-COVID1 and pre-COVID2 to during-COVID-19 than normative expected changes in CRP levels (*p* < 0.003). Changes in CRP levels before and during COVID-19 were not significantly different. (4) Conclusions. These findings highlight the consequential effects of COVID-19, including impairments in sleep, on adolescents with OWOB. CRP levels increased more (~5 mg/L) during COVID-19 than normative expected change.

## 1. Introduction

The coronavirus disease 2019 (COVID-19) pandemic first emerged in December 2019 and spread globally at a rapid pace. Overall, the COVID-19 pandemic disrupted the lifestyles of many families, negatively impacting children’s physical health behaviors and weight status [1]. Sleep habits (i.e., sleep schedules and timing), for example, were disrupted for families during the pandemic [2]. Further, families and children engaged in less physical activity [3], interacted more with screens, and relied on more processed foods [4]. Children and adolescents with overweight or obesity (OWOB) represented a particularly high-risk population for long-term adverse health outcomes associated with the pandemic, as they are at greater risk for the above behavioral health concerns [5].

Adolescent sleep is a health factor sharing a strong relationship with adolescent weight [6]. For example, shorter sleep duration among adolescents is consistently associated with increased risk for OWOB [6]. Specifically, a meta-analysis assessing relations between sleep and obesity among adolescents found that every additional hour of sleep was associated with a 9.0% decreased risk for obesity [6]. This relationship is mediated by numerous factors, including diet quality, increased sedentary behaviors, and mood concerns [7]. Relatedly, sleep was directly impacted by many of the effects of the COVID-19 pandemic. Indeed, in 2020, Becker and colleagues highlighted a range of COVID-19-related risk factors that negatively impact sleep [7]. First, social isolation and remote learning may increase sedentary behaviors and food consumption, directly impacting both sleep and weight status [7]. Second, levels of stress, loneliness, and mood concerns increased among adolescents during the COVID-19 pandemic [7,8]. Such changes also increase risk for insomnia and poor sleep quality [9]. Third, elevated exposure to blue light from increased screen time can disrupt melatonin production—a biological cue for sleep [7]—and decreased exposure to sunlight due to social distancing can disrupt the circadian rhythm [7,10]. Since Becker and colleagues (2020) published this editorial [7], a host of research has emerged to demonstrate that adolescent sleep was in fact negatively impacted by the COVID-19 pandemic [2,11,12,13,14]. Due to the nature of the pandemic, this research primarily relies on self-reported data [12,13,14,15]. However, a more recent study utilizing actigraphy demonstrates similar trends [2]. Many studies utilized brief survey methods to obtain data from large samples [12,13,14] or assessed sleep in typically developing, healthy adolescents [2], which may not be generalizable across groups. Currently, no information is available regarding the specific effects of COVID-19 on sleep in adolescents with OWOB.

Self-reported shifts in bedtimes and wake times are one example of the consequences of COVID-19 [12,13,14]. In a qualitative study, adolescents reported a delayed sleep schedule by about 2 h during the COVID-19 pandemic [14], though estimates vary between studies [12,13,16]. Notably, shifts in these sleep and wake times can be considered temporarily adaptive and may help adolescents more closely align their sleep with their endogenous circadian phase. However, it may later emerge as a barrier when they are expected to return to a typical, in-person routine [7]. Further, decreased structure around bedtimes and wake times may increase variability in sleep timing, which can have negative impacts on sleep.

A second consequence of COVID-19 is poorer sleep efficiency, as indicated by parent-reported adolescent difficulties initiating and maintaining sleep [15]. A review of the research on COVID-19-related sleep difficulties reveals distinct patterns related to age. High school and college students reported poorer sleep during COVID-19 than middle school students [17] while younger adults reported more sleep difficulties than older adults [18]. Consequently, adolescents and emerging adults appear particularly susceptible to sleep changes associated with COVID-19 [15], possibly due to delayed circadian phase and/or greater changes in environmental restraints on sleep scheduling. While these data give us insight into the possible effects of COVID-19 on sleep in adolescents and young adults, confirmatory studies utilizing objective data collection strategies and assessing these effects within vulnerable subpopulations are needed.

Sleep is also strongly associated with C-reactive protein (CRP) [6], a protein involved in the body’s response to inflammation [19], which predicts cardiovascular events and diabetes [19]. Both chronic short sleep duration [20] and long sleep duration [21] are associated with elevated CRP levels in adolescents. Given the abundance of sleep difficulties experienced by adolescents during the COVID-19 pandemic [2,15], it is likely that CRP levels fluctuated similarly. However, while many behavioral changes associated with the COVID-19 pandemic are known to impact CRP levels, no research has examined the relationships between CRP levels and lifestyle changes in the first year of the COVID-19 pandemic. Notably, increased physical activity is associated with decreased CRP levels in adolescents with OWOB [22]. As physical activity decreased among adolescents during the pandemic, especially during virtual schooling periods [3], CRP levels may have been affected. Further, for children and adolescents, decreased levels of CRP are associated with healthy dietary patterns while increased levels of CRP are associated with less healthy dietary patterns [23]. During the first year of the COVID-19 pandemic, access to processed foods and food insecurity increased [24], increasing the prevalence of unhealthy dietary patterns and potentially increasing CRP levels for adolescents who experienced this shift [23]. As noted, sleep also shares a strong relationship with diet quality, and adolescents who report poorer sleep have been found to experience decreased nutrient-rich food intake and increased intake of high-energy, added sugar foods [7]. Thus, the addition of poorer sleep during COVID-19 alongside increased access to processed foods and food insecurity [24] may have a compounding effect on increased CRP levels in adolescents. Given these relationships, and as obesity relates to greater insulin resistance, inflammation, type 2 diabetes, and other cardiovascular diseases [6], adolescents with OWOB present as a high-risk group for a range of adverse physical health outcomes during the pandemic, including elevated CRP levels. This elevated risk may also be directly tied to changes in adolescent sleep.

In a sample of adolescents with OWOB, this study assessed changes in objectively measured sleep and CRP levels during the COVID-19 pandemic. We hypothesized that the COVID-19 pandemic would lead to poorer sleep and elevated CRP levels and that these changes would be related.

## 2. Materials and Methods

### 2.1. Design Overview

This study assessed longitudinal relationships between objectively measured COVID-19 sleep outcomes and CRP levels in adolescents with OWOB. Sleep outcomes, including total sleep time, sleep efficiency, sleep onset latency, total time in bed, wake time, and bedtime, were measured using actigraphy, and CRP levels were measured using fasting blood draws. Analyses compared (1) observed sleep outcomes before COVID-19 with sleep outcomes during COVID-19 (Table 1), (2) observed sleep outcomes during COVID-19 with CRP levels during COVID-19, and (3) CRP levels during COVID-19 with normative expected CRP levels (Table 1). Normative expected CRP levels—or expected CRP levels during the same developmental time period as defined based on pre-COVID-19 norms—were calculated using normative data from the National Health and Nutrition Examination Survey (NHANES) [25] and were demographically and developmentally matched based on each participant’s age, sex, race, and BMI%. Analyses further compared (4) observed change in CRP levels before and during COVID-19 with normative expected change in CRP levels during those same time periods (Table 2), and (5) observed change in CRP levels before COVID-19 with observed change in CRP levels during COVID-19.

All pre-COVID-19 data were collected before March 2020 as part of a larger, ongoing study (NCT02451436) investigating the effects of sleep on insulin sensitivity and body composition among adolescents with OWOB. The present study originated from a previously published research study assessing the effects of COVID-19 virtual learning on TBF% and insulin resistance in adolescents with OWOB. As this study utilized the same study design and dataset, it is important to note similarities in the names of comparison groups and overall study design [26].

The larger study and sub-study were both approved by the Institutional Review Board and are registered on ClinicalTrials.gov (NCT02451436). All participants and their parent/guardian were consented for the larger study, and if they participated in the COVID-19 assessments, they provided additional consent.

For the larger study, participants completed two weeks of actigraphy (measuring sleep outcomes) and two morning blood draws (after fasting for 12 h; measuring CRP levels) at a baseline and 5-week follow-up visit. In October and November of 2020 (~15 months after baseline visit), participants who completed a baseline visit before March 2020 and were currently enrolled in virtual learning were asked to return for an additional follow-up visit. For this visit, participants completed an additional fasting morning blood draw and wore an actigraphy for another week to assess CRP levels and sleep outcomes, respectively, following the COVID-19 pandemic.

### 2.2. Participants

**Figure 1:**
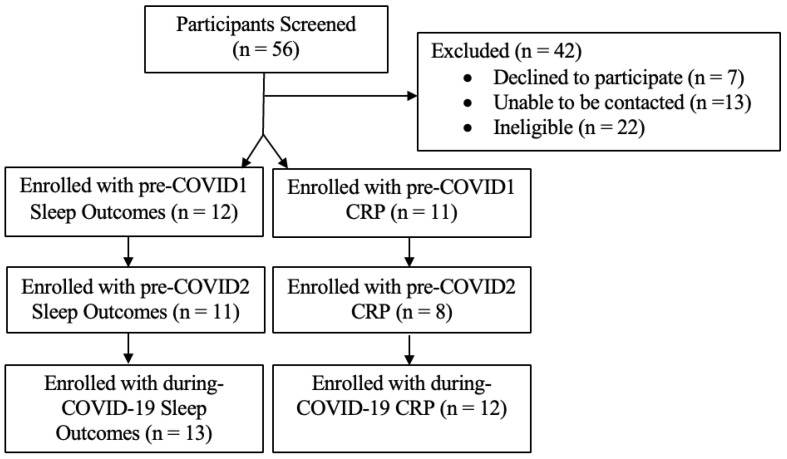
Participant enrollment diagram.

Individuals ages 15–17 years who were classified as having OWOB and had access to a personal smartphone were eligible for the larger study. Adolescents with a BMI% at or above the 85th% were classified as having OWOB. This was determined using the Center for Disease Control and Prevention’s Children’s BMI Group Calculator [27]. Participants that had a sleep disorder or mental illness, were currently enrolled in a weight-loss program, or had a history of bariatric surgery or taking medications that may affect weight or sleep were excluded from the study. See Figure 1 for participant enrollment diagram.

### 2.3. Measurements

Demographics. Participants’ parents/guardians completed a demographics questionnaire that assessed their adolescents’ sex, date of birth, race, and household income.

Sleep Outcomes. Sleep outcomes, including total sleep time, sleep efficiency, sleep onset latency, total time in bed, wake time, and bedtime, were assessed using actigraphy. Participants wore an actigraph on their wrist for one week prior to the baseline (pre-COVID1 sleep outcomes), 5-week follow-up (pre-COVID2 sleep outcomes), and during-COVID-19 visit (during-COVID-19 sleep outcomes).

C-Reactive Protein. Participants’ CRP (mg/L) was assessed at the baseline (pre-COVID1 CRP), 5-week follow-up (pre-COVID2 CRP), and during-COVID-19 visit (during-COVID-19 CRP) via morning blood draws after a 12 h fast. Change in CRP levels before COVID-19 were calculated using pre-COVID1 CRP levels—pre-COVID2 CRP. Change in CRP levels from before to during COVID-19 was calculated using (1) pre-COVID1 CRP—during-COVID-19 CRP and (2) pre-COVID2 CRP—during-COVID-19 CRP.

Normative expected CRP, or expected CRP during the same developmental time period based on pre-COVID-19 norms, was calculated using normative data from NHANES26 and demographically and developmentally matched based on each participants’ age, sex, race, and BMI%. Normative expected change in CRP from before to during COVID-19 was calculated using (1) pre-COVID1 CRP, during-COVID-19 normative expected CRP, and (2) pre-COVID2 CRP, during-COVID-19 normative expected CRP.

Total Body Fat Percent (TBF%). Using enCORE 2011 v15 (sp2) software package and Lunar iDXA instrument (GE Healthcare, Madison, WI, USA), Dual-energy X-ray absorptiometry assessed TBF% during COVID-19.

### 2.4. Data Analyses

A power analysis was conducted in G*Power (v3.1). Based on the previous literature assessing self-reported changes in adolescent sleep during COVID-19 within the U.S., a within-groups effect size of d = 0.89 was utilized [15]. This analysis indicated a sample size of N = 10 to achieve a minimum of 80% power (actual power = 83%) and detect within-subjects differences in adolescent sleep when applying an alpha level of 0.05. An additional power analysis was conducted utilizing a conservative within-groups effect size (d = 0.9) based on the previous literature investigating the effects of changes in sleep on CRP (d = 2.28) [28]. This analysis indicated a sample size of N = 10 to achieve a minimum power of 80% (actual power = 84%) when utilizing an alpha level of 0.05.

To assess the effects of COVID-19 on sleep outcomes, paired samples *t*-tests were used to compare (1) pre-COVID1 and pre-COVID2 sleep outcomes with during-COVID-19 sleep outcomes. Pearson correlations were used to assess (2) the relationship between during-COVID-19 sleep outcomes and during-COVID-19 CRP levels. Linear regression was used to assess the relationship between significant correlations when controlling for during-COVID-19 TBF% (Table 1). To assess the effects of COVID-19 on CRP levels, paired samples *t*-tests were used to compare (3) during-COVID-19 CRP with normative expected during-COVID-19 CRP, (4) change in CRP from pre-COVID1 and pre-COVID2 to during COVID-19 versus normative expected change in CRP during those same time periods, and (5) change in CRP before COVID-19 (pre-COVID2—pre-COVID1) with change in CRP during COVID-19 (during-COVID-19—pre-COVID2). All variables were assessed for normality prior to final analyses (Table 2).

## 3. Results

Fourteen adolescents (Mage = 15.93, 57% male, 71% Black, 21.4% White) with OWOB were included in the final sample (Table 3). The sample size was limited by the number of participants who provided data before COVID-19 and who were enrolled in virtual school during the recruitment phase. However, our power analyses indicated needing a minimum of 10 participants to achieve adequate power. Of the 14 participants, 8 completed the pre-COVID1 and pre-COVID2 morning blood draws and actigraphy before March 2020. Twelve completed the pre-COVID1 actigraphy data before March 2020 (Figure 1). The average duration between pre-COVID1 and during-COVID-19 visits was 15 months.

During-COVID-19 sleep efficiency (N = 11, M = 74.48%, SD = 8.60) was significantly reduced as compared with pre-COVID1 (M = 84.67%, SD = 7.52; t(10) = 4.37, *p* = 0.001, d = 1.26) and pre-COVID2 (M = 84.12%, SD = 5.68; t(9) = 3.80, *p* = 0.002, d = 1.15) sleep efficiency. During-COVID-19 time in bed (M = 514.97, SD = 46.22) was significantly longer than pre-COVID1 time in bed (M = 472.93, SD = 52.27; t(11) = −2.31, *p* = 0.041, d = 0.67). Pre-COVID1 (M = 06:44 AM, SD = 38.87 min; t(11) = −4.47, *p* < 0.001, d = 1.29) and pre-COVID2 (M = 06:16 AM, SD = 49.36 min; t(10) = −5.46, *p* < 0.001, d = 1.65) wake times were significantly earlier as compared with during-COVID-19 wake time (M = 08:00 AM, SD = 48.91 min). During-COVID-19 bedtime (M = 11:19 PM, SD = 30.08 min) was later as compared with pre-COVID2 bedtime (M = 10:21 PM, SD = 62.69 min; t(10) = −2.49, *p* = 0.032, d = 0.78). During-COVID-19 sleep onset latency (M = 68.76 h, SD = 38.40) was significantly longer as compared with pre-COVID1 (M = 29.58 h, SD = 30.51; t(11) = −3.17, *p* = 0.009, d = 0.92) and pre-COVID2 sleep onset latencies (M = 33.76 h, SD = 22.37; t(10) = −3.10, *p* = 0.011, d = 0.93).

During-COVID-19 CRP (M = 5.20, SD = 3.00) was greater than normative expected CRP (M = 0.43, SD = 0.23; t(13) = −5.50, *p* < 0.001, d = 1.59). Increases in CRP from pre-COVID1 to during COVID-19 (M = −1.43, SD = 4.23) and pre-COVID2 to during COVID-19 (M = −1.50, SD = 2.30) were greater than normative expected change in CRP during those same time periods (M = 3.43, SD = 2.80; t(9) = −4.74, *p* = 0.001, d = 1.59; M = 3.37, SD = 2.74; t(7) = −3.88, *p* = 0.006, d = 1.50), respectively. However, change in CRP from pre-COVID1 to pre-COVID2 (M = −0.26, SD = 3.30) and pre-COVID2 to during COVID-19 (M = −1.50, SD = 2.30; t(7) = 1.11, *p* = 0.303) was not significantly different.

During-COVID-19 CRP was positively correlated with during-COVID-19 wake time (r = 0.71, *p* = 0.01) and time in bed (r = 0.72, *p* = 0.008). The significant positive relationship between during-COVID-19 CRP and during-COVID-19 time in bed remained when controlling for TBF% during COVID-19 (t(7) = 4.29, *p* = 0.004).

## 4. Discussion

The present study highlights the consequential health effects of COVID-19 on adolescents with OWOB, including increased time in bed, sleep onset latency, and CRP and decreased in sleep efficiency. A shift in adolescent bedtimes and wake times was also observed such that teens stayed up later and slept in longer during the COVID-19 pandemic. Adolescent time in bed related to adolescent CRP during the COVID-19 pandemic. These findings support our hypotheses that the COVID-19 pandemic would contribute to poorer sleep and elevated CRP levels and that these changes would be related.

The observed changes in sleep timing are in line with the circadian delay that occurs during adolescence and with the past literature, which indicates a general shift toward later wake and bedtimes and extended time in bed for adolescents during the COVID-19 pandemic. However, the magnitude of this shift varies widely between studies and study locations. Previous self-reported research highlights changes in time in bed ranging from an average extension of 5 min in Italy to 12 to 41 min in the United Kingdom [29] and shifts in bedtime and wake time from 36 to 41 min [12], 16 to 45 min, and to 79 min, respectively, in India, Italy, and China [12,13,16]. However, qualitative data from Canada indicate a shift in adolescent bedtime and wake time of approximately 2 h [14], a finding later supported by self-reported data collected by the same research group. Notably, estimates utilizing objective measures of sleep (i.e., actigraphy) have indicated average shifts in bedtime of 22 to 99.6 min and average wake time shifts of 49 to 172 min for adolescents in Australia [30] and the U.S. [15]. Compared with past findings, the present results better align with those estimating larger shifts in adolescent sleep timing, highlighting a 42 min average increase in time in bed and average shifts in bedtime and wake time of 57 and 89.5 min, respectively. Notably, as social distancing guidelines varied widely across countries and even across states within the U.S., it is possible that variations in social distancing guidelines in the southeastern United States contributed to the severe effects of COVID-19 on sleep observed in the present study. Specifically, as social distancing guidelines were less strict in the southeastern United States from where these participants were recruited, participants may have had greater shifts in sleep timing due to the continued availability of social opportunities while also not having to wake as early for school while attending virtually. Further, while the present study supports previous self-reported data regarding the directionality of shifts in sleep during COVID-19, it may also highlight the increased specificity offered by an objective measurement of sleep as compared with self-reported data. However, while some of the limited objective evidence regarding changes in adolescent sleep during COVID-19 align with this theory and indicate greater shifts in sleep [15], this finding is not consistent in other objective research [30]. Thus, it is important to consider other factors that may contribute to this finding, noting that adolescents with OWOB already experienced increased risk of poor sleep prior to COVID-19 [6]. Therefore, the present findings may highlight increased vulnerability of this population to poor sleep outcomes during the COVID-19 pandemic, as this is the first study to investigate these outcomes in adolescents with OWOB specifically.

The observed changes in sleep onset latency and efficiency also trend toward more extreme ends of what was observed in the majority of previous studies [13]. Overall, estimates of average sleep onset latency during the COVID-19 pandemic range from less than 15 min in China [15] and the United Kingdom [29] to approximately 28 min in India, [13] based on self-reported data. Further, adolescent research in the U.S. indicated that sleep onset latency above 60 min was rare, occurring in only approximately 1.9% of participants during the pandemic [15]. Notably, in the present sample, 53.8% of adolescents had objectively measured sleep onset latency greater than 60 min. Further, sleep efficiency was observed to drop from approximately 84% before the COVID-19 pandemic to approximately 74% during the COVID-19 pandemic. This finding, while supported by previous research, is again greater than what was reported in other studies. Past self-reported data indicate parent-reported difficulties in regard to teen initiation and maintenance of sleep during COVID-19 [15] and a decrease in sleep efficiency from 87.9% to 84.15% in U.S. adolescents [2]. However, not all studies have found decreased sleep quality following COVID-19 [14]. Further, while past research estimated that 16.7–35.8% of high school students experienced impairments in sleep efficiency great enough to meet criteria for insomnia (<85%) during COVID-19 [2,17], all but one of the participants in the present study (92.3%) met this criteria during COVID-19 and 58.33% met this criteria prior to COVID-19. These data highlight the importance of understanding the impact of COVID-19 for especially vulnerable groups such as adolescents with OWOB who, as noted above, already experienced increased risk of poor sleep prior to COVID-19 [6]. It is possible that COVID-19 exacerbated this risk through several pathways including (1) removing structural supports for consistent bedtimes and wake times, (2) providing increased opportunity for time in bed outside of sleep, (3) increasing sedentary behavior due to social isolation and remote learning [7], (4) elevating levels of stress, loneliness, and mood concerns [7,8,9], and (5) promoting greater exposure to blue light from increased screen time [7] and decreased exposure to sunlight due to social distancing [7,10]. Overall, it is likely that the present findings regarding extreme shifts in adolescent sleep during COVID-19 are driven by a combination of factors, including the increased vulnerability of individuals with OWOB, social distancing regulations unique to the southeastern U.S., and the specificity offered by objective measurements of sleep.

The present study’s assessment of shifts in adolescent CRP during the pandemic align well with the above findings related to sleep. For teens with OWOB, change in CRP before COVID-19 was similar to normative expected change. However, during the pandemic, CRP for participants in the present study increased by approximately 5 mg/L. This increase holds notable clinical significance as CRP over 3 mg/L is associated with high risk for cardiovascular disease [31]. CRP during COVID-19 was significantly related to during-COVID-19 time in bed, which is expected based on the past literature highlighting the relationship between poor sleep and elevated CRP in adolescents [20]. Additional factors, which may have influenced this outcome, include poorer food choices, increased screen time, increased sedentary time, and decreased physical activity, all of which have been observed during the COVID-19 pandemic [3] and relate to elevated levels of CRP [29,32]. Further, the finding is also supported by previous research demonstrating increased adiposity and insulin resistance in teens during the COVID-19 pandemic [33].

The above findings regarding decreased sleep quality and elevated CRP are concerning for several reasons. Poor sleep in adolescence is related to increased risk for weight gain [34], poorer nutritional food choices [35], impaired cognitive function [36], elevated mood concerns, and heightened risk taking [37]. Elevated CRP in adolescence is also associated with increased risk for many negative health outcomes and correlates including metabolic syndrome, elevated BMI [38], and persistent elevations in inflammation into adulthood [39]. Thus, teens with OWOB who experienced poor sleep and inflammation exacerbations during the COVID-19 pandemic may be at increased risk for further impairments in mental and physical health. Further, there may be differences in outcomes between adolescents with overweight and adolescents with obesity. However, the present study did not assess these differences. Regardless, it is critical that interventions to mitigate these risks are developed and maintained to improve long-term health outcomes within this population. Further, as COVID-19 is still present in the U.S. and there is an ongoing possibility of new variants, it is important to consider how to mitigate outcomes of poor sleep and elevated inflammation if virtual learning or increased social distancing measures are revisited. Such interventions may include ensuring children remain engaged in physical activity, only use the bed for sleep, maintain consistent bedtimes and wake times, and limit their use of screens.

The present study has many strengths. It is the first to assess COVID-19 sleep outcomes and investigate the effects of the COVID-19 pandemic on CRP among adolescents with OWOB. Further, the use of actigraphy allowed for the objective measurement of adolescent sleep outcomes, supporting the observed trends highlighted in self-reported data and the paucity of current research utilizing objective measures. CRP was also measured objectively, and both were assessed before and after COVID-19, allowing for an assessment of changes from before to during COVID-19. Limitations of the study include a small sample. However, the study was adequately powered to detect differences in sleep and CRP from before to during COVID-19 and was strengthened by the use of a within-subjects design and comparisons with demographically and developmentally matched data. Additional limitations include that the effects of puberty on adolescent sleep and CRP were not accounted for, and sleep and CRP are influenced by pubertal development. However, our study found that change in CRP during COVID-19 was greater than expected change in comparison to adolescents of the same sex, age, sex, race, and body mass index. The use of these comparisons during the same time period outside of COVID-19 suggests increased CRP during COVID-19 outside of increased CRP due to the effects of puberty. Additional limitations include the lack of longer term, post-COVID-19 follow-up measures (e.g., 2-year follow-up) and the lack of a control group. Finally, the present study did not assess for potential differences in sleep and CRP outcomes between adolescents with overweight and those with obesity.

Overall, the present study highlights the significant effects of the COVID-19 pandemic and virtual learning on outcomes of sleep and inflammation in adolescents with OWOB in the U.S. The observed results include increases in time in bed, sleep onset latency, and CRP and decreases in sleep efficiency. A shift in adolescent bedtimes and wake times was also observed such that teens stayed up later and slept in longer during the COVID-19 pandemic. Exacerbations in poor sleep and inflammation have the potential to negatively impact both immediate and long-term adolescent health outcomes. Interventions to address the adverse health effects associated with COVID-19 and virtual learning (e.g., schools utilizing virtual learning may consider implementing virtual physical education courses, mandatory technology breaks, virtual healthy eating courses) are critical.

## Figures and Tables

**Table 1:** Outline of sleep outcome analyses.

Analysis	Types of Test	Sleep Outcome	N	Comparison Group 1	Comparison Group 2
Pre-COVID1 sleep outcomes compared with during-COVID-19 sleep outcomes	Paired Samples T-Test	Sleep Efficiency	11	Pre-COVID1 sleep outcomes	During-COVID-19 sleep outcomes
		Total Sleep Time	10		
		Sleep Onset Latency	11		
		Total Time in Bed	10		
		Bedtime	11		
		Wake Time	12		
Pre-COVID2 sleep outcomes compared with during-COVID-19 sleep outcomes	Paired Samples T-Test	Sleep Efficiency	10	Pre-COVID2 sleep outcomes	During-COVID-19 sleep outcomes
		Total Sleep Time	10		
		Sleep Onset Latency	11		
		Total Time in Bed	10		
		Bedtime	11		
		Wake Time	11		
During-COVID sleep outcomes compared with during-COVID-19 CRP	Bivariate Correlations	Sleep Efficiency	8	During-COVID-19 sleep outcomes	During-COVID-19 CRP
		Total Sleep Time	8		
		Sleep Onset Latency	8		
		Total Time in Bed	8		
		Bedtime	8		
		Wake Time	8		

**Table 2:** Outline of CRP analyses.

Analysis	Types of Test	N	Comparison Group 1	Comparison Group 2
During-COVID-19 CRP compared with normative CRP	Paired Samples T-Test	14	(during-COVID-19 CRP)	Normative CRP *
Change in CRP before COVID-19 compared with normative change in CRP	Paired Samples T-Test	10	(pre-COVID1 CRP–pre-COVID2 CRP)	(Normative pre-COVID1 CRP–normative pre-COVID2 CRP) *
Change in CRP during-COVID-19 compared with normative change in CRP	Paired Samples T-Test	8	(pre-COVID2 CRP–during-COVID-19 CRP)	(Normative pre-COVID2 CRP–normative during-COVID2 CRP) *
Change in CRP before COVID-19 compared with change in CRP during-COVID-19	ANCOVA	8	(pre-COVID1 CRP–pre-COVID2 CRP)	(pre-COVID2 CRP–pre-COVID CRP)

* Normative values for CRP were calculated using age, sex, BMI%, and race-matched values.

**Table 3:** Participant demographics.

Characteristics		%
Sex	Male	57.1
	Female	42.9
Household Income	<40,000 USD	7.1
	40,000–59,000 USD	28.6
	60,000–99,000 USD	21.4
	>100,000 USD	42.9
Race	Black	71.4
	White	21.4
	Other	7.1
		**Mean (SD)**
Age (years)		15.93 (0.83)
Sleep Efficiency (percent)	Pre-COVID1	84.67 (7.53)
	Pre-COVID2	84.12 (5.69)
	During-COVID-19	75.71 (9.35)
Total Sleep Time (minutes)	Pre-COVID1	398.58 (40.64)
	Pre-COVID2	395.58 (46.39)
	During-COVID-19	386.81 (50.20)
Sleep Onset Latency (minutes)	Pre-COVID1	29.58 (30.51)
	Pre-COVID2	33.75 (22.37)
	During-COVID-19	64.99 (39.20)
Total Time in Bed (minutes)	Pre-COVID1	472.93 (52.27)
	Pre-COVID2	473.94 (64.88)
	During-COVID-19	513.38 (44.62)
Bed Time (minutes)	Pre-COVID1	1371.10 (58.17)
	Pre-COVID2	1342.01 (62.69)
	During-COVID-19	1402.21 (29.05)
Wake Time (minutes)	Pre-COVID1	404.03 (38.87)
	Pre-COVID2	375.96 (49.36)
	During-COVID-19	480.21 (46.88)
CRP	Pre-COVID1	3.53 (2.90)
	Pre-COVID2	3.76 (2.72)
	During-COVID-19	5.20 (3.05)
TBF%	Pre-COVID1	35.3 (9.8)
	Pre-COVID2	34.3 (12.2)
	During-COVID-19	42.3 (12.1)

## Data Availability

The data presented in this study are available upon request from the corresponding author. The data are not publicly available due to ongoing data analyses of the primary and secondary outcomes.
